# The Role of Natural Antibodies to CC Chemokine Receptor 5 in HIV Infection

**DOI:** 10.3389/fimmu.2017.01358

**Published:** 2017-10-30

**Authors:** Assunta Venuti, Claudia Pastori, Lucia Lopalco

**Affiliations:** ^1^Division of Immunology, Transplantation and Infectious Diseases, DIBIT – San Raffaele Scientific Institute, Milan, Italy

**Keywords:** CC chemokine receptor 5, anti-CC chemokine receptor 5 antibodies, CC chemokine receptor 5 signalosome, HIV infection, HIV protection, CC chemokine receptor 5-based vaccine, CC chemokine receptor 5-based therapy

## Abstract

The CC chemokine receptor 5 (CCR5) is responsible for immune and inflammatory responses by mediation of chemotactic activity in leukocytes, although it is expressed on different cell types. It has been shown to act as co-receptor for the human and simian immunodeficiency viruses (HIV-1, HIV-2, and SIV). Natural reactive antibodies (Abs) recognizing first loop (ECL1) of CCR5 have been detected in several pools of immunoglobulins from healthy donors and from several cohorts of either HIV-exposed but uninfected subjects (ESN) or HIV-infected individuals who control disease progression (LTNP) as well. The reason of development of anti-CCR5 Abs in the absence of autoimmune disease is still unknown; however, the presence of these Abs specific for CCR5 or for other immune receptors and mediators probably is related to homeostasis maintenance. The majority of anti-CCR5 Abs is directed to HIV binding site (N-terminus and ECL2) of the receptor. Conversely, it is well known that ECL1 of CCR5 does not bind HIV; thus, the anti-CCR5 Abs directed to ECL1 elicit a long-lasting internalization of CCR5 but not interfere with HIV binding directly; these Abs block HIV infection in either epithelial cells or CD4+ T lymphocytes and the mechanism differs from those ones described for all other CCR5-specific ligands. The Ab-mediated CCR5 internalization allows the formation of a stable signalosome by interaction of CCR5, β-arrestin2 and ERK1 proteins. The signalosome degradation and the subsequent *de novo* proteins synthesis determine the CCR5 reappearance on the cell membrane with a very long-lasting kinetics (8 days). The use of monoclonal Abs to CCR5 with particular characteristics and mode of action may represent a novel mode to fight viral infection in either vaccinal or therapeutic strategies.

## Introduction

The CC chemokine receptor 5 (CCR5) belongs to G protein-coupled receptors (GPCRs), which represent the largest known superfamily of signal transducers and play functional roles in the response to exposure to light and odor as well as in cellular response to different types of signaling molecules (1). They consist approximately 4% of coded human genome (2) and represent one of the most important and largest groups of targets for therapeutics (3). Among them, the chemokine receptors are responsible for immune and inflammatory responses by mediation of chemotactic activity in leukocytes, even though they are expressed on a wide range of cell types, such as T and B cells, monocytes–macrophages, granulocytes, NK, DC, astrocytes, and neurons, and also on epithelium, endothelium, vascular smooth muscle, and fibroblasts (4–8).

CCR5 has also been implicated in hematopoiesis and it has been demonstrated that it act as co-receptor for the human and simian immunodeficiency viruses (HIV-1, HIV-2, and SIV) either independently of, or together with, the receptor CD4 (9–12). In particular, binding of viral gp120 of HIV-1 to CD4 triggers a conformational change in gp120 itself, which permits its binding to CCR5 and finally the viral entry into the cells (13, 14).

CCR5 is undoubtedly the main HIV-1 and HIV-2 co-receptor, involved in virus entry and cell-to-cell spread (15); interestingly, these R5-tropic viruses (CCR5 dependent strains) are associated with the initial infection (16), while HIV strains using the CXCR4 co-receptor are detected rarely in the early infection (11, 15, 17).

It is well known that chemokine receptor agonists, such as the β-chemokines RANTES (CCL5), MIP-1α (CCL3), and MIP-1β (CCL4), inhibit HIV infection of susceptible cells *in vitro* (18–21).

Interestingly, the number of CCR5 molecules expressed on cell surface is correlated with the levels of viral infection (13) and it has been described a variation of the level of CCR5 molecules among individuals (15), which is due to both environmental and genetic aspects. Indeed, it has been shown that high levels of CCR5, in some developing countries such as Africa, is environmentally driven and it has been hypothesized that it is due to parasitic infections (22). Whereas a CCR5-negative phenotype has been described in either some subjects, which are resistant to HIV infection (exposed to HIV but seronegative subjects, so called ESN) or in Caucasians and in other ethnic groups worldwide; the reduced or absent expression of CCR5 in these populations has been attributed to a genetic mutation, named Δ32, a deletion of 32 base-pair in CCR5 gene that produces a truncated form of the receptor, which is not expressed on the cell membrane (23). Several clinical studies underlined that homozygous mutation affecting the expression of CCR5 confers a total resistance against HIV infection (24–28); whereas heterozygotes for CCR5Δ32 are not associated with complete HIV protection (15) but progress slowly in the infection, most likely due to the reduction of CCR5 levels on the cell surface (29). CCR5Δ32 is spontaneous in 4–18% of Askenazi Jews and European people but it has not been found in Pacific and Asian indigenes (21, 24, 25, 28); this mutation occurs mostly like a heterozygous defect on CCR5 gene (10–20%), with the highest frequencies in Nordic European countries, and only less than 1% is a homozygous mutation, as reported in several study population (24, 25, 30–33). In addition, it has been shown that the frequency of CCR5Δ32 genotype is higher also among ESN and HIV-infected individuals who control disease progression without treatment (so called LTNP) compared to HIV-1 treated seropositive subjects and people from the general population (34, 35). Of note, different levels of CCR5 expression among different individuals do not affect immune functions (36), in fact its absence is not associated with medical dysfunction (37). Nevertheless the prevalence of homozygosity for CCR5Δ32 mutation, which results in the absence of CCR5 expression, has been found increased in either West Nile infected subjects or in tick-borne encephalitis (38, 39) and reviewed in Venuti et al. (21).

More interestingly, anti-CCR5 natural Abs have been discovered and they also showed HIV-blocking properties (40–43).

Overall, several numbers of strategies aimed to the prevention of CCR5 function in the HIV entry has been developed and tested.

## Mechanism of Generation of Anti-Self Antibodies (Abs)

Natural Abs represent the first line of defense against pathogens; they are usually present in human serum as IgG3, IgM, and IgA and are generated in the absence of previous immune activation (44, 45).

The identification of B-1 cells (a subset of B cells), able to produce different self-reactive Abs, has demonstrated the capability of the immune system to interact with self-repertoire (45–47). It has been established that, in human, B cells are able to proliferate and to secrete Abs after exposure to lipopolysaccharide (LPS) from the Gram-negative bacteria membrane independent to the specific B-cell receptor (BCR) (48). Nevertheless, natural human Abs can also cross-react with microbial antigens, thus allowing host protection to pathogen independent of the previous microbial invasion (44).

B-1 cells are detected in the pleural and peritoneal cavity and represent the first line of defense, but they are present in the spleen and bone marrow as well, in which they secrete a higher proportion of circulating natural Abs (40, 45). The activation status of B-1 cells is BCR independent (49) and after their fast redistribution from the body cavities, B-1 cells are able to differentiate and to secrete abundant amounts of IgM and/or IgA (50).

The partial differentiation of B-1 cells and their ability to respond rapidly are fundamental for the Abs production to elucidate host protection to pathogens infection *via* mucosal surfaces and blood. In fact, the production of natural IgM at a steady state by B-1 cells represents a relevant protection against pathogen replication before the development of the antigen-specific response (40, 44, 45, 51, 52).

Many functions have been proposed for natural Abs such as a first line role in host defense and also a regulative part in homeostasis maintenance (40, 45, 53, 54). In addition, B-1 cells produce IgM that stimulate B-2 cells to elicit IgG (45, 55, 56), but they can also lead to induce the IgA production in response to antigen stimulation especially in the serum or in the intestinal lamina propria (40, 57).

Since the Eighties, when the AIDS was first described, several signals of autoimmune dysfunction were reported in subjects infected with HIV, such as B cell altered pathway, with production of high quantity of Abs and also of anti-cell Abs (58–60). These abnormalities, at the beginning, were related to HIV-vs-host activity but other pieces of evidence suggested that some anti-cell Abs may be considered like a host-vs-HIV reactions. Actually, it was shown that some broadly neutralizing human Abs produced during the HIV infection were autoreactive (61). The finding led to suppose that immunotolerance mechanisms represent a disadvantage for these types of Abs (62, 63). Notably, the studies regarding the follow-up of HIV patients treated with three broadly neutralizing Abs, established that only one of them exhibited a low level of *in vivo* autoreactivity, while autoimmune-related adverse events were not detected in the study (64).

Many healthy donors displayed the presence of natural reactive Abs specific for CCR5 in several pools of immunoglobulins (41). Interestingly, different types of HIV-blocking Abs have been isolated from several cohorts of either ESN or HIV-infected individuals (40). The reason of development of anti-CCR5 Abs in the absence of autoimmune disease is still unknown; however, the presence of these Abs specific for CCR5 or for other immune receptors and mediators probably is related to homeostasis maintenance (40). Virus-induced alterations of self antigens can provide an increase of either auto-immunogenic proteins and the corresponding auto-Abs. Host factors itself, or other concomitant or latent viral infections, could activate these perturbations in the host cells, leading to conformational changes in host receptors and to remodeling from a self protein to a non-self antigenic epitope, as reviewed by Lopalco (40).

## CCR5 and Its Related Abs

CCR5 shows a classic structure composed of seven transmembrane domains with N-terminus and three extracellular loops (ECL1, 2, and 3), which have immunogenic properties. The two longer domains (N-terminus and ECL2) are recruited for HIV binding (65–67). Its preferential ligands are MIP-1α, MIP-1β, and RANTES and the binding of these molecules could interfere sterically with the viral envelope protein (Env) gp120 of HIV binding resulting in an inhibition of viral infection (15). An alternative model of protection is that ligand-induced chemokine receptor internalization eliminates the co-receptor from the cell surface (68); obviously, these two mechanisms are not mutually exclusive.

Anti-CCR5 natural Abs were found also in individuals with Δ32 mutation, sexual partners of subjects who were wild type for CCR5 gene, thus suggesting that CCR5 can be considered as an alloantigen (40, 42, 69, 70). Moreover, hemophilic patients subjected to continuous blood transfusions, ESN and LTNP show Abs to CCR5 directed specifically to the first external loop (ECL1) (21, 29, 41–43, 69, 71–75); these natural Abs have been identified in serum and also in other biological fluids, such as semen, cervicovaginal secretion and saliva in subject with different genetic background (75).

The majority of anti-CCR5 Abs is directed to HIV binding site (N-terminus and ECL2) of the receptor. Conversely, Abs to ECL1–CCR5, which induce a long-lasting internalization of the receptor (29), are capable to block HIV infection in either CD4+ T lymphocytes or epithelial cells, this latter one through transcytosis, which mimics mucosal transmission (76) and this mechanism differs from that induced by all the other ligands directed to CCR5 (40). First of all, the natural Abs recognize ECL1 whereas CCR5 agonists specifically bind to the ECL2 of CCR5. Second and more important, the long-lasting internalization of CCR5 with natural anti-CCR5 Abs seems to be a unique mechanism not demonstrated for other CCR5 modulating molecules so far. Indeed, by using monoclonal antibodies (mAbs) that recognize the N-terminus and the second loop of CCR5, it has been shown a differentially modulation of receptor activity; thus suggesting that each CCR5 extramembrane region can display different properties (65, 77, 78).

A clinical study, related to the presence and the activity of Abs to ECL1 in the sera of some LTNP, clearly demonstrated that the loss of these Abs observed during the follow-up of these subjects was significantly associated with the clinical progression of the disease (29). Moreover, in another studies, a total of 206 Asian and Caucasian ESN subjects have been tested for the presence of anti-CCR5 Abs directed to ECL1 and 9% resulted positive (43, 75, 79), similar percentage (9.8%) have been found in different cohorts of HIV seropositive subjects (total subjects 336) (29, 80), although only in LTNP anti-CCR5 Abs have been associated with resistance and showed anti HIV property *in vitro* (29, 81). Strikingly, anti-CCR5–ECL1 Abs resulted HIV protective only when they were directed to a conformational epitope within ECL1 loop (43, 75). A total of 325 healthy controls have even analyzed as well but none resulted positive for anti-CCR5 Abs, thus suggesting that these Abs could be elicited by low levels of viral antigenic stimulation; that could explain why these Abs have been found in ESN and LTNP people but not in subjects who were not exposed to HIV or progressed and developed AIDS. Another hypothesis could be that anti-CCR5 Abs are elicited during other antigenic stimulations (different from HIV), which induce alterations of self-repertoire, thus eliciting anti-self responses. Finally, the priming due to endogenous retroviral proteins, which share homology with HIV env protein, could elicit in some HIV-exposed subjects a specific immune response.

Of note, these ECL1 specific Abs do not induce alteration in immune functions, as demonstrated by healthy subjects with anti-CCR5 Abs (45) or by elicited anti-CCR5 Abs in animal models such as mice and macaques (82–84) as further specified in the section of CCR5 immunization as vaccination strategy.

The ECL2 domain represents the binding site for both HIV and chemokines, so the Abs that recognize this site can prevent chemokine binding and/or signaling (66), although N-terminus is specific for viral binding only. For example, 2D7 is one of the most potent mAb directed to ECL2 that blocks HIV-1 entry into CD4 T cells, but not the transcytosis carried out with epithelial cells (66, 76, 85). An anti-CCR5 mAb named PRO140 is a humanized mAb that targets a conformational epitope between N-terminus and ECL2 and it deeply blocks viral entry (86). Another fully human IgG4 mAb with a strong activity against various HIV-1 isolates is CCR5mAb004 (87).

A recent study has demonstrated for the first time that the region designated as the membrane-proximal region (MPR), between the N-terminus and the ECL1, is important for HIV-1 infections (16). In fact, the Abs directed to this epitope block the infection of R5-tropic HIV-1 without affecting X4-tropic strain; furthermore, the substitution of MPR with the equivalent region of CCR2b, CXCR4, or CCR3 significantly abrogates viral infection (16). Both these findings provide an argument against the possible use of a target therapy with CCR5-specific Abs.

## Endocytosis and *de novo* Synthesis of CCR5 with Natural Anti-CCR5 Abs

Ligands binding to CCR5 leads to conformational changes, which include desensitization and internalization (88). Two major mechanisms of rapid receptor regulation have been distinguished, specifically homologous (agonist-specific) and heterologous (agonist-nonspecific) desensitization, and both mechanisms are really important in fine tuning leukocyte responses (89, 90). Homologous desensitization requires phosphorylation of the receptor binding mediated by members of the GPCR kinases (GRK) family (91). This in turn leads to the association of β-arrestin1/2 with the receptor and to desensitization *via* uncoupling of the receptor and G protein (77, 92); in particular, β-arrestins bound physically with the receptors and initiate endocytosis through clathrin-coated vescicles and also act as scaffold proteins in crosstalk with other signaling pathways (93). Conversely, heterologous desensitization is traditionally defined as a state of cellular refractoriness to different agonists after receptor phosphorylation sites different from GRK mediated by second messenger-activated protein kinases, such as PKC (90).

CCR5 internalization can also induce a different second pathway, which recruits caveolae and it is independent of clathrin-coated pits. Caveolae are microdomains able to be internalized under precise conditions or in a controlled manner (13, 94).

It is well known that, after endocytosis, the GPCR proteins are also classified in receptors that are recycled, slowly or rapidly, to the cell membrane after their resensitization and those that should be degraded (77, 95–97). CCR5 is usually recycled after desensitization (4): after stimulation with natural ligands, CCR5 is internalized into the trans-Golgi network (TGN) *via* the endosome recycling compartment (ERC) (98) and, when the resensitization process is complete, it can return to the cell surface (4, 98). However, rare examples of post-endocytic sorting for GPCRs mediated by ligands have been reported (77, 99–101).

Bönsch and colleagues have recently shown that different ligands of the same GPGR are able to induce different phosphorylation pathways, which may be a relevant factor for the interaction with β-arrestins (77, 102). In addition, ligands trigger a characteristic short-term kinetics of CCR5 internalization, which transiently involves β-arrestins with consequent rapid recycling or degradation on the cell membrane; conversely, natural anti ECL1-CCR5 Abs induce a specific long-lasting kinetics of CCR5 internalization (29) with the recruitment of an ERK1-mediated pathway (70, 77). Of note, a hitherto unrecognized mechanism of CCR5 modulation mediated by G-protein-dependent ERK1 was comprehensively reported; in particular, natural anti-CCR5 Abs led to activation of ERK1 which is localized predominantly in the cytosol and it interacts directly with the CCR5 protein, thus inducing the degradation of CCR5 with a consequent *de novo* synthesis (70); the re-expression of CCR5 on the cell surface needs several days (70). This finding is actually important for the design of suitable microbicide or therapeutic tool that could inhibit HIV infection for several days after application by using a specific molecule able to induce long-lasting internalization and degradation of CCR5.

Furthermore, it is largely reported that GPCRs, considering the stability of interaction with β-arrestins after agonist stimulation, can be functionally divided into two general classes: (i) “Class A” receptors, such as β2 adrenergic receptor (β2AR), develop transient complexes with β-arrestins transiently ubiquinated and with weak activation of ERK1/2; by contrast, (ii) “Class B” receptors, such as vasopressin V2 receptor (V_2_R), develop tight receptor–β-arrestins complexes, regulated by its constant ubiquitination and a durable activation of ERK1/2 which is located mainly into the endosomes. Endosomes complexes containing activated GPCRs, activated and ubiquitinated β-arrestins and phosphorylated ERK are called “signalosome” (77, 102, 103). In fact, it is well understood that the ubiquitination status of β-arrestin has a relevant role for its interaction with proteins responsible for endocytosis (e.g., clathrin) and for signaling (e.g., ERK1/2), and influences temporal and spatial dissociation of the complex (104–108). Overall, CCR5 is classified as a “Class A” receptor, but stimulation with anti-CCR5 Abs lead to the translation into a very long-lasting Class B type (77, 102, 106).

Very recently, it has been published the different ability of two RANTES analogous (5P14 and PSC) to induce the development of stable complexes between CCR5 and β-Arrestin1. Briefly, PSC-RANTES is able to induce a long-duration of recruitment of β-Arrestin1 to CCR5 compared to 5P14-RANTES, which elicits a temporary recruitment. Notably, the experiments have been carried out and the results assessed at short time only (50 min) (1). Therefore, it is possible to determine the fate of the internalized receptor by the aid of specific CCR5-ligands, suggesting that the stability of ligand-induced receptor–arrestin complexes has a crucial role in the sorting mechanism (1, 77).

**Figure 1:**
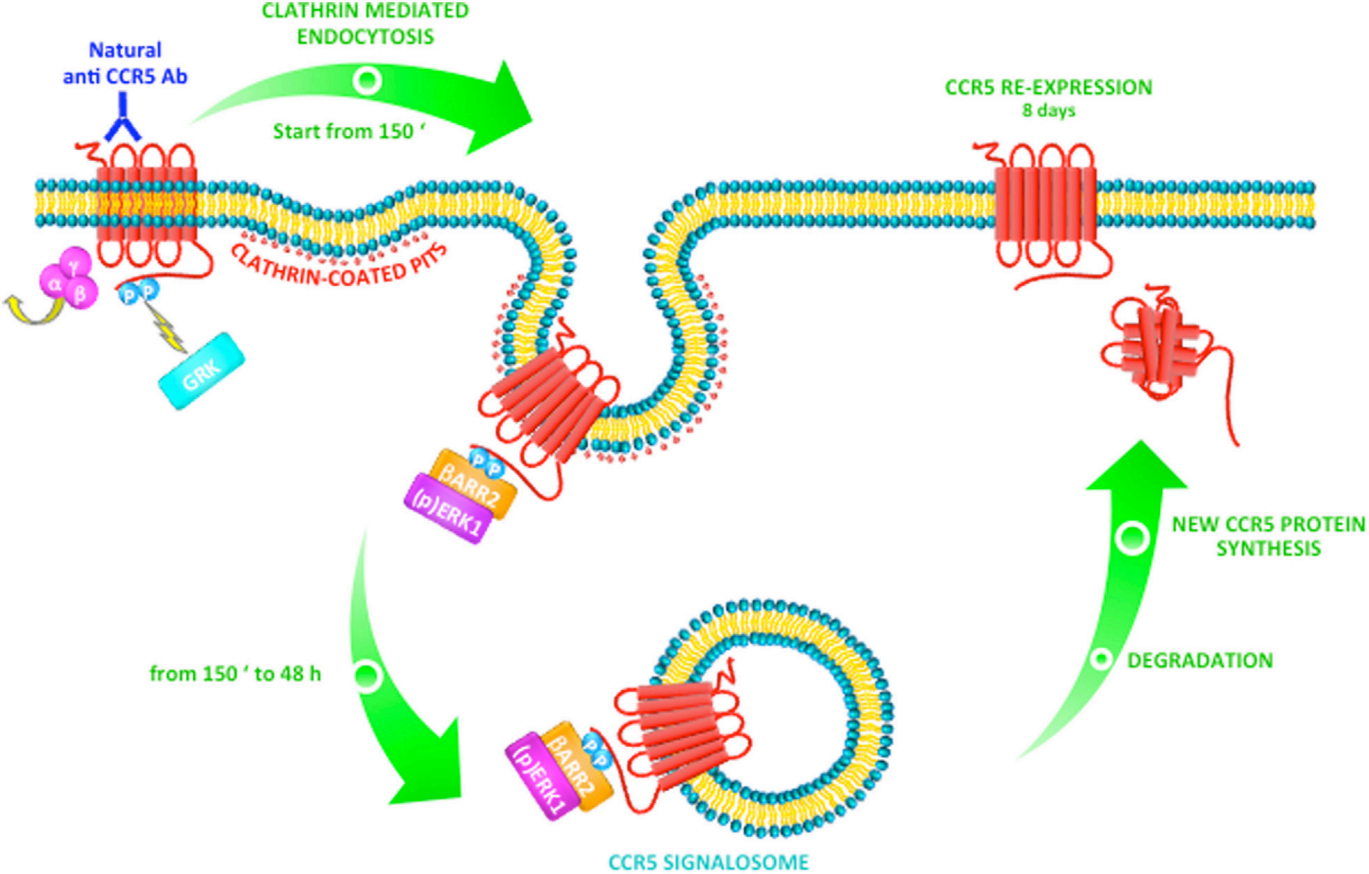
Natural anti-CCR5 antibodies (Abs) to ECL1 triggers a Class B CCR5 trafficking pathway in T cells. After stimulation with anti-CCR5 Abs, the CCR5 receptor associates with G protein and G protein-coupled receptor kinases (GRKs) trigger receptor phosphorylation. β-arrestin1/2 can initiate desensitization (at 150 min) with consequent internalization of CCR5 by clathrin-coated pits. In particular, activated CCR5 together with β-arrestin2 is accumulated into protein complexes and induces the activation and retention in the cytoplasm of MAP kinase ERK1. These events determine the formation of a CCR5 signalosome with β-arrestin2 and ERK1 into the cytosol, which remains stable from 150 min up to 48 h. The signalosome could be targeted for degradation with consequent *de novo* synthesis of the proteins complex (CCR5, β-arrestin2, and ERK1). As a consequence, CCR5 reappears on the cell surface with long-lasting kinetics (8 days).

In a very relevant way, these published data underline that the binding of natural Abs induces modifications in CCR5 signaling, which leads ligand-induced post-endocytic sorting in a very long-lasting Class B trafficking (77). Furthermore, in T cell, anti-CCR5 Abs that recognize ECL1 are able to induce a CCR5-negative phenotype, ERK1-mediated, by the strong support of β-arrestin2 (as shown in Figure 1); otherwise, it is possible that this mechanism could be specific for T cells only (77, 109).

## Induction of Anti-CCR5 Abs as Vaccination Strategy

Published data, obtained in mice and macaques, demonstrate the capability of either anti-CCR5 Abs to display HIV-blocking properties or vaccines against CCR5 to prevent the problem of virus variability and viral escape (82, 110–113). Accordingly, the development of Abs as functional inhibitors of CCR5 is the big goal that could be reached, since Abs can provide protection by causing very low toxicity (113). Several groups have investigated the possibility to use *in vivo* Abs specific to CCR5 (82, 83, 111, 112, 114–116). Interestingly, when a long-term intranasal immunization was performed, it has elicited specific IgA and IgG in both mucosal secretions and sera of the immunized mice. Such systemic and mucosal Abs induce a CCR5-negative phenotype on both peripheral and mucosal cells, thus blocking HIV replication *in vitro* (111). In accordance with this result, the use of ECL1–CCR5 peptide, chimeric-generated in the context of the capsid protein of Flock House Virus, elicits Abs able to induce CCR5 internalization and re-expression with a very slow kinetics which needs 4 weeks after immunization to be recovered (82). Furthermore, in a subsequent study, it has been published that the substitution of amino acids within ECL1 in position 95 and 96 elicited Abs, which induced stronger long-lasting internalization of CCR5, whereas amino acid substitutions in position 92, 98 and 99 abrogated biological activity of such Abs (112), thus highlighting the importance of the epitope in driving different trafficking pathway. Moreover, in a recent study performed in mice, several aspects of anti-CCR5 immunization, including the use of all the extramembrane domains of CCR5 have been tested, to better understand the ideal schedule to reach long-lasting and strong immune responses. Interestingly, ECL1 and ECL2 showed stronger responses compared to the N-terminus; they achieved nearly complete CCR5 downregulation, and they blocked HIV infection (82). In addition, in this study was not observed any immune dysfunction in T cell responses or histopathological alterations in organs and tissues in relation to the presence or the induction of Abs specific for CCR5. The possibility of long-term toxicity and any functional impact of anti-CCR5 Abs needs additional studies; however, the findings showed in this latter study are supported by other published studies, where no adverse events were reported in CCR5-immunized macaques after 3 years of follow-up (84). In addition, it has recently published that the prophylactic immunization of macaques with virus-like particle specific for two CCR5 regions is safe and immunogenic and is capable to reduce highly virus replication in a subset of the animals (83). On the other hand, Bogers and colleagues used an immunization approach to target both virus and CCR5 (three extracellular peptides of CCR5, an N-terminal HIV gp120 fragment generated in transgenic plants and recombinant SIV p27) (117); this strategy of vaccination showed a significant block of the virus infection by eliciting good serum and vaginal quantity of Abs (117). More recently, Peabody et al. demonstrated that the immunization with recombinant vectors, which enable the CCR5–ECL2 region to recreate its native conformation, overcomes the issue of tolerance and induces the appropriate immune response (118).

Although several strategies aimed at inducing a CCR5-negative phenotype to prevent HIV-1 entry, the earlier immunization studies in macaques observed little or no protection against SIV challenge (116, 118), probably due to poor selection of CCR5 antigen or to the correct peptide sequence in the wrong conformation. Indeed, it has previously demonstrated that immunization with ECL1 domain, in a linear conformation, does not elicit serological Abs responses that bind to the native molecule (111) and, moreover, in macaques, the immunization with ECL2 in its native conformation induces immune responses with expected properties (84). Nevertheless, Chain and colleagues have recently defined a new linear epitope of CCR5 within the N-terminus domain recognized by two independently produced mAbs; in particular, they found that RoAb13 Ab is capable to bind to both linear peptide and native form of the epitope and the sulfation of tyrosines at CCR5 N-terminus enhanced its binding to the peptide (119). RoAb13 has been previously reported to block HIV infection (120) but also blocks migration of monocytes after the chemokine binding to CCR5 or in the presence of inflammatory macrophage conditioned medium (119).

A significant challenge in the design of anti-CCR5 Abs is that they must be purely “blocking Abs” that either bind to the epitope in such a way to occlude the viral receptor or Abs binding results in receptor internalization. The most effective anti-pathogen Abs are able to engage host defense mechanisms, such as Complement or ADCC (Antibody-Dependent Cell-mediated Cytotoxicity), thus resulting protective against HIV infection (121) although these functions could result in inhibition of the effectiveness of immune responses. Moreover, as reported by Pastori et al., it is possible to elicit the production of murine serum anti-ECL1–CCR5 Abs at levels 300-fold greater than those found in humans and that the quantity of murine CCR5-specific immunoglobulins reached 50% of total Igs (82). It is noteworthy that such HIV-1 blocking Abs are present in serum and mucosal fluids from subjects with different genetic backgrounds (75), thus suggesting that it is possible to elicit these Abs in subjects coming from both developing as well as developed countries. In addition, an individual who received a stem cell transplant from a CCR5-negative donor, for acute myeloid leukemia treatment, is believed to be the only patient to have been cured of HIV (119, 122).

## Anti-CCR5 Abs in the Immune-Prophylaxis Against HIV Infection

The Abs can prevent viral infection by several mechanisms of action: (1) can directly block virus attachment to the cell by leading the Abs to bind either virus or receptor and/or co-receptor on host cells; (2) can block fusion at cell surface at the post-binding/pre-fusion state as well (87). For reducing the development of viral escape variant, it has been highly considered to target the conserved cellular receptors, such as CCR5, for treatment of HIV infection. In particular, as HIV needs the presence of one co-receptor in dependence of the strain (CCR5 and/or CXCR4) in association with the receptor CD4, mAbs against cellular proteins have been developed and are being tested in clinical trials. A humanized mAb directed to CD4, named ibalizumab, exert an antiviral property not inhibiting the binding of gp120 but by a post-binding conformational effects, which prevents the interaction between CD4-gp120 and CXCR4 or CCR5 (123, 124). Three clinical trials have been reported, which underlined its efficacy (87). For sure, one emerging therapy is based on the use of CCR5-specific Abs; in particular, CCR5mAb004 appears safe and effective in the reduction of viral load when tested in clinical trials (87). Interestingly, another study involving the mAb PRO140 showed virologic suppression without blocking the response of the receptor to chemokines; however, the highest tolerated dose of this mAb has not been determined, proposing a substantial margin of safety for PRO140 in dependence of the site of administration (87, 125). In all these clinical trials, the use of anti-CCR5 Abs did not induce any alterations in other lymphocyte functions, thus confirming their safety.

Of note, the use of Abs instead of chemokines or classical antiretroviral therapy could reduce the complication related to drugs resistance and also the unwanted interactions with redundant CCR receptors. For example, ST6 is a Fab fragment obtained from a mAb specific for a unique sequence of N-terminus CCR5 and it was engineered in a single-chain antibody (scFv) fused with an ER retention peptide; the usage of such scFv by intracellular immunization was able to downregulate the receptor from cell membrane both in macaques and in human cells, whereas the expression of CXCR4 was not affected. Moreover, the modified cells were not infected with R5-HIV (126). In a subsequent study, it has been demonstrated that transformed primary T cells, with a CCR5 intrabody (an Ab that binds its receptor at intracellular level), were resistant to HIV infection (21). Finally, scFvs directed to CCR5 were utilized, as well, to lead viral pseudotyped lentiviral vectors to cells that express CCR5 (127).

Very recently emerged the evidence that combinations of HIV-blocking Abs will likely be more effective that single one as reviewed by Margolis (128). Alternatively, the bio-engineering, which generates Abs either with different specificities (129) or anchored to target cells (130), has given a proof of concept to generate more potent HIV-blocking Abs.

## Other Strategies Aimed at Blocking HIV Infection Through CCR5

Anti-CCR5 strategies include also the utilization of small molecule drugs, such as Maraviroc, which binds in the transmembrane regions of CCR5 and it is a functional antagonist that prevents CCR5 signaling from cell surface and even if it is currently in clinical trials (131), it has been approved for use in many jurisdictions.^1^ Nevertheless, there is low enthusiasm to utilize it as front-line therapy in HIV-infected patients (23), thus it is currently in use in HIV treatment-multiexperienced patients only (132). Moreover, HIV-1 escape mutants to Maraviroc have been described and reviewed by Harada and Yoshimura (133).

Since the discovery that natural ligands of CCR5 (RANTES, MIP-1α, and MIP-1β) show anti-HIV activity (1, 19, 86, 134, 135), a large numbers of modified analogs have been tested due to their short half-lives (<10 min) (134, 136) but no one has been tested in human clinical trial due to low antiviral activity *in vivo*. The most promising described so far was PSC-RANTES that shows several non-natural, non-coded structures in the N-terminal region (137, 138). It displays an important inhibition of HIV entry, CCR5 dependent, *in vitro* (137) and also a full protection against R5-tropic SHIV infection in a macaque vaginal challenge model (139); although this high potency *in vitro*, it requires high concentration to give protection in macaques (138, 139). Considering that it is capable to induce an intracellular sequestration of CCR5 longer than RANTES, it could be helpful for topical HIV prevention (140). Using a strategy based on phage display, Gaertner and collaborators obtained three different modified PSC-RANTES, which exhibit only natural amino acids: 6P4-RANTES, which prolongs the intracellular sequestration of CCR5; 5P12-RANTES has no detectable G protein signaling and does not bring about receptor sequestration; and 5P14-RANTES, which induces the internalization of CCR5 with no detectable G protein-linked signaling activity (138). Another relevant RANTES derivative is named AOP-RANTES and it was obtained by first generating an aldehyde-like group at the NH2-terminus of RANTES and then reacting with aminooxypentane; it is able to block R5-tropic strain infection on macrophages *in vitro* (141). AOP-RANTES induces >90% downregulation of cell membrane expression of CCR5 on monocytes/macrophages, lymphocytes and inhibits CCR5 recycling on cell surface whereas RANTES does not (142).

As HIV entry process requires expression of both CCR5 and CD4 on cell membrane, receptor- and co-receptor-mimetic peptides (143, 144) have been proposed as an alternative strategy to block HIV entry but, as for chemokines, no one has been already tested in human clinical trial.

**Figure 2:**
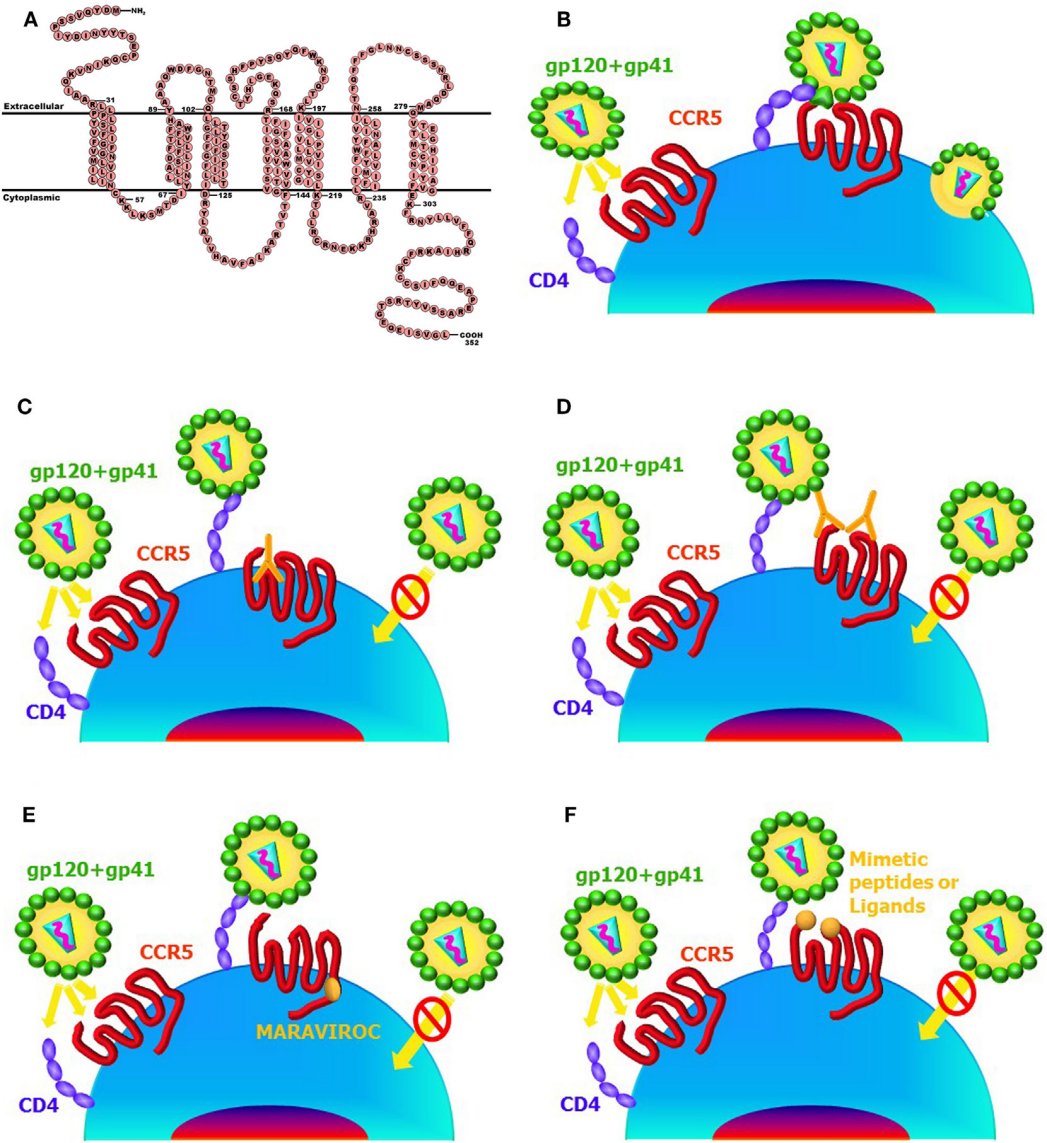
Main anti-CCR5 strategies working at target cell surface. CCR5 protein sequence (https://en.wikipedia.org/wiki/CCR5#/media/File:CCR5_Primary_Protein_Sequence.png) **(A)**. HIV entry process **(B)**. Natural antibodies (Abs) to CCR5 bind to the ECL1 domain, induce long-lasting internalization of the receptor, and block HIV infection **(C)**. Abs to either N-terminus or ECL2 domains of CCR5 compete with HIV-binding site and interfere with HIV infection **(D)**. CCR5 allosteric modulators, such as MARAVIROC, do not allow HIV entry **(E)**. Ligands (such as RANTES or its modified analogous) bind to the ECL2 or mimetic peptides bind to the either N-terminus or ECL2 and interfere with HIV entry process **(F)**.

A summary of the immunologic approaches that use CCR5 as target to block HIV transmission/infection is showed in Figure 2.

Hematopoietic stem cell transplant using a CCR5Δ32 donor led to the only known cure of HIV-1 infection (122, 145) and T cells treated with engineered nucleases that introduce mutations at the CCR5 locus are resistant to HIV (146–150), accelerating ongoing efforts to develop gene editing- and cell-based therapeutic agents for HIV (15, 151, 152).

Another promising method of gene editing is the use of CRISPR/Cas9 system (Clustered Regularly interspaced palindromic repeats sequences) to target human cells for the disruption of CCR5 gene, otherwise the off-targeting is still a major limit to be overcome (153–155). Furthermore, DNA binding proteins, for example, the transcription activator-like effectors (TALEs), which are vegetal proteins, have been used *in vitro* and showed effects similar to those obtained with engineered nuclease (156).

Zinc finger nucleases (ZFNs) are other common and versatile DNA binding proteins utilized in several cell types. In addition, CCR5–ZFN-modified autologous CD4+ T lymphocytes have been used in a phase I clinical trial and this approach resulted safe (149).

To shutdown CCR5 expression, several RNA-based technologies have been used also with good results, such as RNA silencing (siRNA), antisense RNAs targeting different cellular and viral genes or ribozymes with catalytic activity (157–159); in particular, pseudotyped lentivirus and adenoviruses vectors have been used with good results for transducing siRNA-coding sequence into the cells. In the same way to that described for gene editing, off-targeting activity and over-expression of antisense RNA could cause a toxic effect (160) and could activate innate immune response as well (161).

## Conclusion

The incidence of natural allo- or auto-responses in healthy people, without symptoms or signals of autoimmune disease, and also the capability of eliciting and maintaining strong and long-lasting HIV-blocking Abs in animal models, suggests that some autoimmune mechanisms could be positively utilized to give a better protection or a higher response to HIV in HIV-exposed individuals and in HIV-positive subjects. Allo- and auto-immune responses could allow a new key to analyze HIV tricks in immune escape and offer unexploited strategies to fight HIV with its own arms. CCR5 is the most important co-receptor in the early stages of infection, and half or more of all infected individuals move to AIDS harboring only CCR5 (R5)-tropic viruses. Epidemiology studies clearly established that CCR5 plays a crucial role in the transmission and pathogenesis of HIV *in vivo*.

As in CCR5-defective individuals were not found inflammatory and immune alterations or disfunctions, CCR5 has been defined as a redundant molecule in humans (12, 141, 162, 163), and as the variability of HIV *env*, CCR5 has become a relevant target to generate drugs and immune modulatory molecules to block HIV transmission and subsequent infection.

Overall, these findings together with the data reported for *in vivo* (clinical trials) and *in vitro* (laboratory findings) studies support the view that CCR5 could represent an excellent target to fight HIV and a good alternative to classical antiviral approaches, although it should be taken into account the concomitant geographical location of *CCR5*Δ*32* and other pathologies, such as West Nile infection or tick-borne encephalitis.

The development of a sterilizing vaccine capable to prevent HIV infection totally is the highest and the most expected effort, still far from being reached. Over the past 30 years, there has been a huge global effort to develop an effective prophylactic vaccine against HIV/AIDS. This is a significant challenge since no previously licensed vaccine in current use has been designed without the presence of a significant “convalescent population,” i.e., patients who have been patently infected and demonstrated subsequent clearance of the pathogen. Such a patient population usually supplies critical information for characterizing adaptive immunological responses associated with “protection.” One of the main reasons of failure in developing an effective AIDS vaccine could be the mainstream concept that the most relevant information derive from studying the immune responses in patients who have not cleared the virus. Thus, the design of a CCR5-based vaccine, which takes advantage of data generated in a small but significant clinical cohorts of individuals such as ESN or LTNP could represent an excellent target to generate new vaccination strategy, as these subjects represent a sort of vaccinated/cured subjects and this protective status can be induced and reproduced in all subject. It is relevant underline that natural anti-CCR5 Abs reproduce a protective status similar to that one observed for Δ32 mutation, although an approach based on CCR5 vaccine in individuals who can contract HIV infection may be a more possible and safe goal compared to gene therapy, taking into account the HIV epidemiology and the trouble of implementing CCR5 gene therapy in people living in developing countries.

Nowadays, there are many antiviral drugs used in therapy but the most related problem is the development of drug-resistant strain of virus that invalidates the positive effects obtained with the therapy utilized. Conversely, the possibility of using monoclonal Abs as therapy, with particular characteristics and mode of action, may represent a novel mode to fight viral infection disease. Overall, Abs show low toxicity together with high specificity and versatility.

It is well known that the first effective treatment of infectious disease was the “serum therapy” (administration of hyperimmune sera from immunized animals or human donors) and only after the discovery of antibiotic therapy in association with the development in vaccine design, this treatment was abandoned for mostly of infections (87, 164, 165).

The possibility of usage of Abs in clinical practice was opened from the opportunity of generate and manipulate Abs with different specific epitope recognition, such as the mAbs (87). In fact, in the last years, mAbs have begun a new class of clinical drug utilized in inflammatory diseases, immunology, and oncology; only their development for infection treatment is going slowly.

Strategies aimed to prevent infection, such as usage of condoms, represent another effective line of defense to fight the HIV epidemic. However, social and ethnic “barriers” impede effective protection of many people. Therapeutic Abs to CCR5 could offer an alternative for primary prevention of HIV and their availability would greatly empower women/men to protect themselves and their partners. Indeed, Abs formulated as a topical product could control the disease without affecting social and procreation aspects. In addition, proceeding directly at the HIV transmission level, the passive immunotherapy approach will help to prevent and reduce both further infection and disease incidence, respectively.

Other strategies involve ART (Anti Retroviral Therapy), which is a strong treatment program utilized to suppress HIV viral replication and the progression of HIV disease. The typical regimen combines three or more different drugs, such as nucleosidic or non-nucleosidic inhibitors of reverse transcriptase, protease, and integrase inhibitors. ART is the only current available treatment for HIV patients and it is being used in many developing countries with the help of WHO.^2^ Nevertheless, it has limitations in terms of high cost, intolerance, bad compliance, and insurgence of resistance (166, 167).

For this reason, a new strategy has emerged to identify blocking Abs against the HIV receptors or co-receptors, either as active-immunizations such as a vaccine or passive-immunizations such as the use of CCR5-based immuno-prophylaxis.

Interestingly, natural human Abs that recognize the ECL1 of the receptor induce a long-lasting internalization of CCR5 by triggering the recruitment of β-arrestin2; this event induces the accumulation of the two proteins (CCR5 and β-arrestin2) into the cytoplasm and leads to the activation of ERK1, which is retained into the cytosol as well. This stable CCR5 signalosome persists into the cells at least 48 h; after that, it may be targeted for degradation with consequent *de novo* synthesis of the proteins complex and, consequently, CCR5 reappears on the cell membrane with long-lasting kinetics (8 days) (70, 77). This particular mechanism could be used for designing molecules that work synergistically for stable maintenance of the signalosome into the cells and for driving the complex to degradation; thus permits to reach a long-lasting CCR5 disappearance from cell membrane which could inhibit HIV infection for a long time.

These findings may support the discovery of innovative therapeutic tools where CCR5 is an important player for microbial control and/or elimination (168) and as well as for the regulation T cell function in autoimmune diseases, such as rheumatoid arthritis, type 1 diabetes, multiple sclerosis (169), and in tumorigenesis (170, 171).

## Author Contributions

AV wrote the review, CP performed the figures and revised the whole manuscript. LL wrote the review and supervised the figures and the whole text.

## Conflict of Interest Statement

The authors declare that the research was conducted in the absence of any commercial or financial relationships that could be construed as a potential conflict of interest.

## Footnotes

^1^https://aidsinfo.nih.gov/guidelines/search/1/CELSENTRI/0.

^2^WHO | Ten years in public health 2007-2017. WHO Available at: http://www.who.int/publications/10-year-review/dg-letter/en/.
